# A hybrid of long short-term memory neural network and autoregressive integrated moving average model in forecasting HIV incidence and morality of post-neonatal population in East Asia: global burden of diseases 2000–2019

**DOI:** 10.1186/s12889-022-14321-3

**Published:** 2022-10-19

**Authors:** Ying Chen, Jiawen He, Meihua Wang

**Affiliations:** Respiratory Medicine Department, XiXi Hospital of HangZhou (Affiliated HangZhou XiXi Hospital, Zhe Jiang University School of Medicine), No.2 Hengbu Road, Liuxia Street, Xihu District, Hangzhou, 310000 Zhejiang Province China

**Keywords:** Long short-term memory neural network model, Autoregressive integrated moving average model, Human immunodeficiency virus. Incidence and mortality, East Asia

## Abstract

**Background:**

To forecast the human immunodeficiency virus (HIV) incidence and mortality of post-neonatal population in East Asia including North Korea, South Korea, Mongolia, Japan and China Mainland and Taiwan province.

**Methods:**

The data on the incidence and mortality of HIV in post-neonatal population from East Asia were obtained from the Global Burden of Diseases (GBD). The morbidity and mortality of post-neonatal HIV population from GBD 2000 to GBD 2013 were applied as the training set and the morbidity and mortality from GBD 2014 to GBD 2019 were used as the testing set. The hybrid of ARIMA and LSTM model was used to construct the model for assessing the morbidity and mortality in the countries and territories of East Asia, and predicting the morbidity and mortality in the next 5 years.

**Results:**

In North Korea, the incidence and mortality of HIV showed a rapid increase during 2000–2010 and a gradual decrease during 2010–2019. The incidence of HIV was predicted to be increased and the mortality was decreased. In South Korea, the incidence was increased during 2000–2010 and decreased during 2010–2019, while the mortality showed fluctuant trend. As predicted, the incidence of HIV in South Korea might be increased and the mortality might be decreased during 2020–2025. In Mongolia, the incidence and mortality were slowly decreased during 2000–2005, increased during 2005–2015, and rapidly decreased till 2019. The predicted incidence and mortality of HIV showed a decreased trend. As for Japan, the incidence of HIV was rapidly increased till 2010 and then decreased till 2015. The predicted incidence of HIV in Japan was gradually increased. The mortality of HIV in Japan was fluctuant during 2000–2019 and was slowly decreased as predicted. The incidence and mortality of HIV in Taiwan during 2000–2019 was increased on the whole. The predicted incidence of HIV during was stationary and the mortality was decreased. In terms of China Mainland, the incidence and mortality of HIV was fluctuant during 2000–2019. The predicted incidence of HIV in China Mainland was stationary while the mortality was rapidly decreased.

**Conclusion:**

On the whole, the incidence of HIV combined with other diseases in post-neonatal population was increased before 2010 and then decreased during 2010–2019 while the mortality of those patients was decreased in East Asia.

## Background

Human immunodeficiency virus (HIV) is a tremendous challenge to the society with 37.7 million people living with HIV, 1.5 million new HIV infections and 680,000 deaths from AIDS-related causes that occurred in 2020 all over the world [1, 2]. HIV has many routes of transmission including mother-to-child transmission (MTCT) [3]. Women with HIV infection at childbearing age are associated with a risk of MTCT during pregnancy and breastfeeding [4]. Most children were infected with HIV via MTCT and the Joint United Nations Program on HIV/AIDS (UNAIDS) estimated that there were 160,000 new HIV infections among children in 2018 [5]. It is well documented that the MTCT rate during pregnancy or postpartum period varies from 15 to 45% without prophylaxis [6]. Maternal HIV infection is associated with a significantly increased risk of infant death with 20% of untreated perinatally infected infants dying within 3 months and 48% dying within 1 year [7]. This indicated the high mortality rate due to HIV in the post-neonatal (1 month to 1 year) population. Effective surveillance and accurate prediction of HIV are essential for the control and outcome improving in post-neonatal population with HIV. Previously, growing numbers of researchers conducted studies on forecasting the incidence of HIV [8–10]. But these studies were performed in all individuals. To date, there was no data on forecasting the HIV incidence and mortality of post-neonatal population.

Recently, various machine learning (ML) and deep learning (DL) were extensively proposed and applied for various aspects including ground water storage change [11], climate [12, 13], permafrost distribution [14], air pollution [15], weed detection [16], botnet detection [17]. Disease forecasting is vital for the prevention of diseases and mathematical models are important tools for understanding of epidemics [18]. Previously, several mathematical models were also applied in forecasting the incidence of infection diseases including HIV [18, 19]. The autoregressive integrated moving average (ARIMA) model proposed by Box and Jenkins in the early 1970s, is a classical model based on linear theory to predict future tendency using the past and present data of time series [20]. The ARIMA model comprehensively considers the change of time trend, periodicity and other factors to achieve better prediction effect, and it is widely applied in predicting the incidence of diseases [21, 22]. The long short-term memory (LSTM) model is a novel prediction tool and a kind of Recurrent Neural Networks (RNN) using the multi-layer and complex neural networks close to the real values and a backward propagation algorithm to continually shrink the fitting error [23]. LSTM can skip the complex mathematical modelling and solving processes, which is suitable to solve the problem of correlation in time series [24]. Increasing evidence suggested that the LSTM model is a useful method for forecasting the incidence of diseases [25, 26].

In this study, we planned to forecast the HIV incidence and mortality of post-neonatal population in East Asia including North Korea, South Korea, Mongolia, Japan and China Mainland and Taiwan via constructing ARIMA and LSTM prediction model based on the data from the Global Burden of Disease (GBD) Study 2000–2019.

## Methods

### Data source

The data on the incidence of HIV in post-neonatal population from East Asian were obtained from the GBD 2000-GBD 2019 (https://www.healthdata.org/gbd/2019). The GBD is a multinational collaborative research study estimating the disease burden of 204 countries and territories [27]. It is an ongoing study updated annually including the incidence, prevalence, mortality, years of life lost (YLLs), years lived with disability (YLDs), and disability-adjusted life-years (DALYs) of 369 diseases and injuries, for two sexes in 204 countries and territories from 1990 to 2019 [27]. The GBD provides information for researchers and clinicians in discovering the latest knowledge and the current status of health for populations and countries worldwide [28]. In this study, HIV burden from 2000 to 2019 in post-neonatal population from East Asian was selected to analyze the time trends of incidence and mortality during 2010–2019 as well as the predicted trends of incidence and mortality in these patients.

### Construction of the hybrid of ARIMA and LSTM model

The hybrid of ARIMA and LSTM model was constructed as follows: ARIMA time series model was employed to predict the morbidity and mortality of post-neonatal HIV population using the Statsmodels package in Python. The formula of the model was Φ(B)(1 − B) dXτ = θ(B)ετ. For construction of the model, firstly, time-series data were collected from the GBD, and the data were plotted to identify whether the time series was stationary. The d order difference operation was conducted in non-stationary time series to convert them into a stationary time series. The autocorrelation function analysis and the partial autocorrelation function analyses were used to identify the possible values of *P*. The parametric tests not statistically significant (*P* > 0.05) in the model were excluded, and the residual tests showed the non-white noise sequence using the Box–Jenkins Q test.

LSTM model was used to train and predict the residual values of ARIMA model using the Torch package in Python. For the construction of LSTM model, the raw data were divided into the testing set (data from 2014 to 2019), and the training set (data from 2000 to 2013). The training samples were used to build the model and to discover potential relationships in the data, and the test samples were used to evaluate the predictive power of the model constructed by the training set. The series of LSTM models were constructed using N values which were the time steps. The model with the lowest mean square error (MSE) was considered the optimal model. Finally, the incidence and mortality of HIV in post-neonatal population from East Asian were predicted by the optimal model for the assignment of the lowest MSE.

The final hybrid of ARIMA and LSTM model was calculated by adding the results of residual values of ARIMA time series model and ARIMA model predicted by LSTM model. The time complexity of the model was O (n^2^), and the space complexity was O (1).

### Statistical analysis

The annual percent changes (APC) for morbidity and mortality were calculated using the Joint Point. The morbidity and mortality of post-neonatal HIV population from 2000 to 2013 were applied as the training set and the morbidity and mortality from 2014 to 2019 were used as the testing set. The hybrid of ARIMA and LSTM model was used to construct the model for assessing the morbidity and mortality in the countries and territories of East Asia, and predicting the morbidity and mortality in the next 5 years. The hybrid of ARIMA and LSTM model was constructed using Python V3.7.4. The performance of the model was evaluated by measuring its persistence including mean absolute error (MAE), the mean squared error (MSE), and root mean square error (RMSE).

## Results

### The incidence of HIV in post-neonatal population from East Asia during 2000–2019

**Fig. 1:**
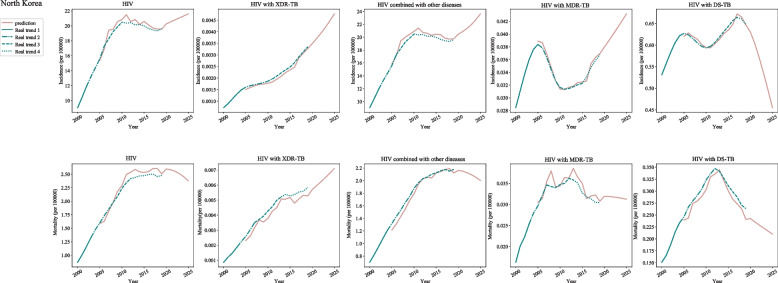
The predicted and actual incidence and mortality of HIV in North Korea

In post-neonatal population from North Korea, the incidences of HIV were increased during 2000–2002, 2002–2006, and 2006–2010, and decreased during 2010–2019. The respective APCs were 14.7 (95%CI: 11.8–17.6), 9.8 (95%CI: 8.4–11.2), 4.4 (95%CI: 3.1–5.8) and − 0.7 (95%CI: − 0.9--0.5). As for HIV with extensive drug-resistant tuberculosis (XDR-TB), the incidence was increased during 2000–2004 (APC = 20.3, 95%CI: 18.9–21.8), 2004–2011 (APC = 3.2, 95%CI: 2.5–3.9) and 2011–2019 (APC = 7.0, 95%CI: 6.6–7.4). The incidences of HIV combined with other diseases were increased during 2000–2002 (APC = 14.7, 95%CI: 11.8–17.6), 2002–2006 (APC = 9.8, 95%CI: 8.4–11.2) and 2006–2010 (APC = 4.4, 95%CI: 3.1–5.8). The incidences of HIV with multidrug-resistant TB (MDR-TB) were increased during 2000–2005 (APC = 6.3, 95%CI: 5.5–7.1), 2010–2015 (APC = 0.6, 95%CI: − 0.5-1.6) and 2015–2019 (APC = 3.5, 95%CI: 2.4–4.6). The incidences of HIV with drug-susceptible TB (DS-TB) were increased during 2000–2004 (APC = 4.2, 95%CI: 3.9–4.5) and 2011–2017 (APC = 2.0, 95%CI: 1.8–2.2) (Fig. 1) (Table 1).

**Table 1:** The incidence and mortality of HIV in post-neonatal population from East Asia during 2000–2019

	Lower Endpoint	Upper Endpoint	APC	Lower CI	Upper CI	Test Statistic (t)	Prob > |t|
North Korea incidence							
HIV	2000	2002	14.7*	11.8	17.6	12.3	< 0.001
	2002	2006	9.8*	8.4	11.2	16.8	< 0.001
	2006	2010	4.4*	3.1	5.8	7.8	< 0.001
	2010	2019	−0.7*	−0.9	− 0.5	−7.1	< 0.001
XDR-TB	2000	2004	20.3*	18.9	21.8	33.8	< 0.001
	2004	2011	3.2*	2.5	3.9	10.8	< 0.001
	2011	2019	7.0*	6.6	7.4	35.8	< 0.001
Combined with other diseases	2000	2002	14.7*	11.8	17.6	12.3	< 0.001
	2002	2006	9.8*	8.4	11.2	16.8	< 0.001
	2006	2010	4.4*	3.1	5.8	7.8	< 0.001
	2010	2019	−0.7*	−0.9	−0.5	−7.1	< 0.001
MDR-TB	2000	2005	6.3*	5.5	7.1	19	< 0.001
	2005	2010	−4.6*	−5.6	−3.6	−10.4	< 0.001
	2010	2015	0.6	−0.5	1.6	1.2	0.245
	2015	2019	3.5*	2.4	4.6	7.6	< 0.001
DS-TB	2000	2004	4.2*	3.9	4.5	30.6	< 0.001
	2004	2011	−0.8*	−1	−0.7	− 11.8	< 0.001
	2011	2017	2.0*	1.8	2.2	21.1	< 0.001
	2017	2019	−1.5*	− 2.4	−0.5	−3.5	< 0.001
North Korea mortality							
HIV	2000	2003	15.4*	14.1	16.8	28.2	< 0.001
	2003	2007	8.7*	7.5	10	16.5	< 0.001
	2007	2012	5.5*	4.8	6.3	16.7	< 0.001
	2012	2019	0.2	−0.1	0.5	1.3	0.213
XDR-TB	2000	2003	28.8*	24.5	33.3	16.9	< 0.001
	2003	2007	16.2*	12.3	20.2	10	< 0.001
	2007	2013	6.9*	5.3	8.5	9.9	< 0.001
	2013	2019	1.5*	0.4	2.7	3	< 0.001
Combined with other diseases	2000	2004	14.8*	13.8	15.8	34.7	< 0.001
	2004	2011	7.2*	6.7	7.7	32.8	< 0.001
	2011	2019	1.2*	0.9	1.5	8.4	< 0.001
MDR-TB	2000	2003	15.5*	11.6	19.6	9.4	< 0.001
	2003	2007	7.3*	3.7	11.1	4.6	< 0.001
	2007	2013	0.6	−0.9	2.2	0.9	0.392
	2013	2019	−2.9*	−4.1	−1.8	−5.7	< 0.001
DS-TB	2000	2004	12.2*	11	13.3	24.1	< 0.001
	2004	2012	5.1*	4.7	5.6	24.9	< 0.001
	2012	2019	−4.2*	−4.6	−3.8	−21.2	< 0.001
South Korea incidence							
HIV	2000	2003	82.6*	70.5	95.5	19.2	< 0.001
	2003	2011	10.3*	8.3	12.4	11.7	< 0.001
	2011	2019	− 3.6*	−5.1	−2.2	−5.4	< 0.001
XDR-TB	2000	2004	17.1*	15.9	18.2	35.4	< 0.001
	2004	2014	−0.1	−0.4	0.2	−1	0.344
	2014	2017	5.1*	1.8	8.5	3.6	0.006
	2017	2019	−3.1*	−6.2	0	−2.3	0.049
Combined with other diseases	2000	2003	82.6*	70.5	95.5	19.2	< 0.001
	2003	2011	10.3*	8.3	12.4	11.7	< 0.001
	2011	2019	−3.6*	−5.1	−2.2	−5.4	< 0.001
MDR-TB	2000	2005	3.4*	2.3	4.6	6.8	< 0.001
	2005	2013	−7.2*	−7.8	−6.5	−25	< 0.001
	2013	2017	−0.3	−2.8	2.2	−0.3	0.766
	2017	2019	−6.7*	− 11.3	−1.9	−3.1	0.013
DS-TB	2000	2005	−2.4*	−2.9	− 1.8	−9.4	< 0.001
	2005	2009	14.3*	12.9	15.8	23.6	< 0.001
	2009	2017	−0.4*	−0.8	−0.1	−2.9	0.016
	2017	2019	−5.3*	− 7.7	−2.9	−4.8	< 0.001
South Korea mortality							
HIV	2000	2010	8.2*	5.1	11.4	5.8	< 0.001
	2010	2019	−4.7*	−7.9	−1.3	−3	0.009
XDR-TB	2000	2004	36.5*	23.1	51.4	6.4	< 0.001
	2004	2019	−2.9*	− 4.2	−1.5	− 4.5	< 0.001
Combined with other diseases	2000	2003	82.6*	70.5	95.5	19.2	< 0.001
	2003	2011	10.3*	8.3	12.4	11.7	< 0.001
	2011	2019	−3.6*	−5.1	−2.2	−5.4	< 0.001
MDR-TB	2000	2004	27.4*	14.9	41.2	5.1	< 0.001
	2004	2009	−13.7*	−22.2	−4.4	−3.1	0.008
	2009	2019	−6.0*	−8.3	−3.6	−5.3	< 0.001
DS-TB	2000	2010	8.9*	6	11.9	6.7	< 0.001
	2010	2019	−4.5*	−7.5	−1.4	−3.1	0.007
Mongolia incidence							
HIV	2000	2006	−6.6	−15.3	3	−1.5	0.153
	2006	2015	7.7*	1.2	14.7	2.6	0.024
	2015	2019	−26.6*	−38.8	−12	−3.7	0.003
XDR-TB	2000	2004	9.0*	4.7	13.4	4.8	< 0.001
	2004	2010	1.6	−1.3	4.5	1.3	0.242
	2010	2016	16.4*	13.1	19.7	12.1	< 0.001
	2016	2019	−14.4*	− 19.7	− 8.8	−5.6	< 0.001
Combined with other diseases	2000	2003	8.8*	2.5	15.5	3.2	0.011
	2003	2006	−9.6	−19.8	1.9	−1.9	0.09
	2006	2016	8.1*	6.9	9.3	16.1	< 0.001
	2016	2019	−32.7*	− 36.6	−28.5	−14.9	< 0.001
MDR-TB	2000	2010	−4.8*	−5.6	− 4.1	−14.2	< 0.001
	2010	2014	11.3*	5.8	17.1	4.8	< 0.001
	2014	2017	1.3	−8.4	12.1	0.3	0.776
	2017	2019	−23.6*	−31	−15.5	−6	< 0.001
DS-TB	2000	2002	−7.6*	−13.2	−1.7	−2.9	0.018
	2002	2010	−17.2*	− 17.9	− 16.5	−51	< 0.001
	2010	2017	1.9*	0.8	3	4	0.003
	2017	2019	−26.3*	−30.8	−21.5	−11	< 0.001
Mongolia mortality							
HIV	2000	2003	−0.8	−6.3	5.1	−0.3	0.772
	2003	2007	−8.7*	−13.9	−3.3	−3.6	0.006
	2007	2016	7.8*	6.5	9.2	13.6	< 0.001
	2016	2019	− 32.6*	−36.4	−28.6	− 15.5	< 0.001
XDR-TB	2000	2010	−1.6*	−2.8	− 0.5	−3	< 0.001
	2010	2016	13.8*	9.9	17.8	8.2	0.012
	2016	2019	−28.3*	−33.7	− 22.6	−9.4	< 0.001
Combined with other diseases	2000	2003	8.8*	2.5	15.5	3.2	0.011
	2003	2006	− 9.6	−19.8	1.9	−1.9	0.09
	2006	2016	8.1*	6.9	9.3	16.1	< 0.001
	2016	2019	−32.7*	−36.6	−28.5	−14.9	< 0.001
MDR-TB	2000	2010	−9.6*	−10.7	−8.5	−17.7	< 0.001
	2010	2016	8.4*	4.6	12.3	4.9	< 0.001
	2016	2019	−31.8*	−37	−26.1	−10.4	< 0.001
DS-TB	2000	2010	−19.1*	−20.2	−18.1	−34.4	< 0.001
	2010	2016	2.4	−1.5	6.5	1.4	0.2
	2016	2019	−30.8*	−36.5	−24.5	−9.3	< 0.001
Japan incidence							
HIV	2000	2004	−0.5	−2.5	1.6	−0.5	0.6913
	2004	2012	8.1*	7.2	9.1	19.9	< 0.001
	2012	2016	−15.8*	−18.5	−12.9	−11.7	< 0.001
	2016	2019	1.3	−2	4.7	0.9	0.412
XDR-TB	2000	2002	− 6.3*	−9.1	−3.6	−5	< 0.001
	2002	2010	−13.2*	−13.5	−12.9	− 81.5	< 0.001
	2010	2015	4.7*	3.8	5.7	11.3	< 0.001
	2015	2019	−18.4*	−19.1	− 17.6	−49.3	< 0.001
Combined with other diseases	2000	2004	−0.5	−2.5	1.6	−0.5	0.613
	2004	2012	8.1*	7.2	9.1	19.9	< 0.001
	2012	2016	−15.8*	− 18.5	− 12.9	− 11.7	< 0.001
	2016	2019	1.3	−2	4.7	0.9	0.411
MDR-TB	2000	2002	−12.1*	−18.9	−4.8	−3.7	0.005
	2002	2009	−22.4*	− 23.4	− 21.3	−42.4	< 0.001
	2009	2015	−2.7*	−4.4	−0.9	−3.4	0.008
	2015	2019	−21.3*	−23.3	−19.3	− 21.4	< 0.001
DS-TB	2000	2002	−5.0*	−7.9	−2	−3.7	0.004
	2002	2010	−11.3*	− 11.6	−10.9	−65.4	< 0.001
	2010	2015	−3.7*	−4.6	−2.8	−8.7	< 0.001
	2015	2019	− 22.6*	− 23.4	− 21.8	−59.3	< 0.001
Japan mortality							
HIV	2000	2007	2.7	−0.6	6.2	1.8	0.098
	2007	2019	−3.2*	−4.6	−1.8	−4.8	< 0.001
XDR-TB	2000	2011	−2.4*	− 4.4	−0.4	− 2.6	0.021
	2011	2019	3.3	0	6.8	2.1	0.051
Combined with other diseases	2000	2007	4.6*	1	8.3	2.8	0.017
	2007	2019	−3.2*	−4.7	−1.7	−4.5	< 0.001
MDR-TB	2000	2010	−10.4*	−12.5	−8.3	− 10	< 0.001
	2010	2019	−1.9	−4.5	0.8	−1.5	0.158
DS-TB	2000	2019	−2.5*	−3.3	−1.6	−6.3	< 0.001
Taiwan incidence							
HIV	2000	2012	11.1*	9.8	12.4	19.8	< 0.001
	2012	2015	−12.8	−28.3	6	− 1.5	0.152
	2015	2019	2.1	−4	8.7	0.7	0.469
XDR-TB	2000	2005	6.4*	5.8	7	23.7	< 0.001
	2005	2010	−5.7*	−6.4	−4.9	−15.8	< 0.001
	2010	2017	0.8*	0.3	1.2	4	0.003
	2017	2019	4.2*	1.5	7	3.5	0.006
Combined with other diseases	2000	2012	11.1*	9.8	12.4	19.8	< 0.001
	2012	2015	−12.8	−28.3	6	−1.5	0.152
	2015	2019	2.1	−4	8.7	0.7	0.469
MDR-TB	2000	2006	−4.4*	− 4.6	− 4.2	−46.2	< 0.001
	2006	2010	−12.9*	−13.5	− 12.3	−47.8	< 0.001
	2010	2017	−4.1*	−4.3	−3.9	− 43.2	< 0.001
	2017	2019	0.5	−0.8	1.8	0.9	0.398
DS-TB	2000	2019	−2.3*	− 3.1	−1.4	−5.5	< 0.001
Taiwan mortality							
HIV	2000	2011	8.6*	6	11.3	7.3	< 0.001
	2011	2019	−3.1	−6.8	0.8	−1.7	0.111
XDR-TB	2000	2019	−0.7	−1.6	0.3	−1.5	0.164
Combined with other diseases	2000	2011	12.9*	9.9	16	9.7	< 0.001
	2011	2019	−3.6	−7.7	0.6	−1.8	0.09
MDR-TB	2000	2019	−6.9*	−7.9	−5.9	−13.7	< 0.001
DS-TB	2000	2019	−2.3*	−3.1	−1.4	−5.5	< 0.001
China mainland incidence							
HIV	2000	2007	5.9*	4.8	7	12.2	< 0.001
	2007	2013	−4.2*	−5.9	−2.5	−5.5	< 0.001
	2013	2016	0.8	−6.9	9.1	0.2	0.826
	2016	2019	−12.6*	−16	−9	−7.7	< 0.001
XDR-TB	2000	2006	0.1	−0.7	0.9	0.3	0.806
	2006	2010	−10.8*	− 12.9	−8.6	−10.7	< 0.001
	2010	2014	−5.9*	−8.2	−3.6	−5.7	< 0.001
	2014	2019	6.3*	5.1	7.4	12.8	< 0.001
Combined with other diseases	2000	2007	5.9*	4.8	7	12.2	< 0.001
	2007	2013	−4.2*	−5.9	−2.5	−5.5	< 0.001
	2013	2016	0.8	−6.9	9.1	0.2	0.826
	2016	2019	−12.6*	−16	−9	−7.7	< 0.001
MDR-TB	2000	2005	−8.8*	−10.1	− 7.5	− 15	< 0.001
	2005	2010	−15.4*	−17	− 13.7	−19.1	< 0.001
	2010	2014	−11.3*	−14.1	−8.5	− 8.7	< 0.001
	2014	2019	2.2*	0.8	3.6	3.5	0.006
DS-TB	2000	2002	−3.1	−7.3	1.3	−1.6	0.148
	2002	2010	−6.2*	−6.7	−5.6	−24.2	< 0.001
	2010	2013	−2.5	−6.7	1.9	−1.3	0.231
	2013	2019	−0.1	−0.8	0.7	−0.3	0.784
China mainland mortality							
HIV	2000	2008	3.8*	2.2	5.5	5.1	< 0.001
	2008	2019	−0.6	−1.6	0.3	−1.4	0.191
XDR-TB	2000	2007	0.1	−1.5	1.8	0.2	0.881
	2007	2014	−11.2*	− 13	−9.3	− 12.4	< 0.001
	2014	2019	−2.9*	−5.6	−0.2	−2.3	0.037
Combined with other diseases	2000	2008	7.0*	5.2	8.8	8.5	< 0.001
	2008	2019	0.4	−0.7	1.4	0.7	0.482
MDR-TB	2000	2007	−8.6*	−10	−7.3	−13.3	< 0.001
	2007	2014	−16.0*	−17.5	− 14.4	−20.3	< 0.001
	2014	2019	−7.0*	−9.3	−4.7	−6.4	< 0.001
DS-TB	2000	2009	−4.6*	−5.5	−3.7	−10.5	< 0.001
	2009	2019	−7.8*	−8.5	−7	−21.4	< 0.001

*APC* Annual percent changes, *XDR-TB* Extensive drug-resistant tuberculosis, *MDR-TB* Multidrug-resistant TB, *DS-TB* Drug-susceptible TB

**Fig. 2:**
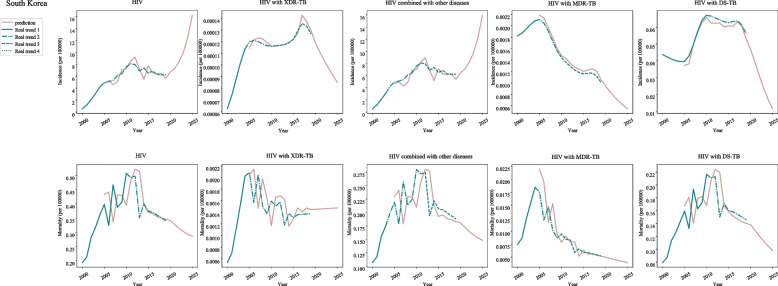
The predicted and actual incidence and mortality of HIV in South Korea

In post-neonatal population from South Korea, the incidences of HIV were increased during 2000–2003 (APC = 82.6, 95%CI: 70.5–95.5) and 2003–2011 (APC = 10.3, 95%CI: 8.3–12.4). In terms of HIV with XDR-TB, the incidences were increased during 2000–2004 (APC = 17.1, 95%CI: 15.9–18.2) and 2014–2017 (APC = 5.1, 95%CI: 1.8–8.5). As for HIV combined with other diseases, the incidences were increased during 2000–2003 (APC = 82.6, 95%CI: 70.5–95.5) and 2003–2011 (APC = 10.3, 95%CI: 8.3–12.4). With regard to HIV with MDR-TB, the incidences were increased during 2000–2005 (APC = 3.4, 95%CI: 2.3–4.6), and decreased during 2005–2013 (APC = -7.2, 95%CI: − 7.8--6.5), 2013–2017 (APC = -0.3, 95%CI: − 2.8-2.2) and 2017–2019 (APC = -6.7, 95%CI: − 11.3--1.9). The incidences of HIV with DS-TB were increased during 2005–2009 (APC = 14.3, 95%CI: 12.9–15.8), and decreased during 2000–2005 (APC = -2.4, 95%CI: − 2.9--1.8), 2009–2017 (APC = -0.4, 95%CI: − 0.8--0.1) and 2017–2019 (APC = -5.3, 95%CI: − 7.7--2.9) (Fig. 2) (Table 1).

**Fig. 3:**
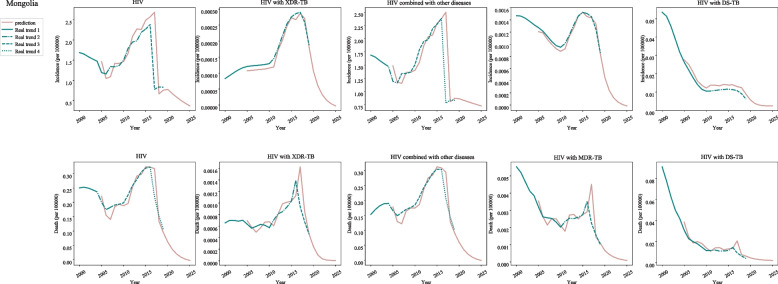
The predicted and actual incidence and mortality of HIV in Mongolia

In post-neonatal population from Mongolia, the incidences of HIV were increased during 2006–2015 (APC = 7.7, 95%CI: 1.2–14.7), and decreased during 2000–2006 (APC = -6.6, 95%CI: − 15.3-3) and 2015–2019 (APC = -26.6, 95%CI: − 38.8--12). The incidences of HIV with XDR-TB were increased during 2000–2004 (APC = 9.0, 95%CI: 4.7–13.4), 2004–2010 (APC = 1.6, 95%CI: − 1.3-4.5) and 2010–2016 (APC = 16.4, 95%CI: 13.1–19.7), and decreased during 2016–2019 (APC = -14.4, 95%CI: − 19.7--8.8). The incidences of HIV with combined with other diseases were increased during 2000–2003 (APC = 8.8, 95%CI: 2.5–15.5) and 2006–2016 (APC = 8.1, 95%CI: 6.9–9.3), and decreased during 2003–2006 (APC = -9.6, 95%CI: − 19.8-1.9) and 2016–2019 (APC = -32.7, 95%CI: − 36.6--28.5). The incidences of HIV with MDR-TB were increased during 2010–2014 (APC = 11.3, 95%CI: 5.8–17.1) and 2014–2017 (APC = 1.3, 95%CI: − 8.4-12.1), and decreased during 2000–2010 (APC = -4.8, 95%CI: − 5.6--4.1) and 2017–2019 (APC = -23.6, 95%CI: − 31--15.5). The incidences of HIV with DS-TB were increased during 2010–2017 (APC = 1.9, 95%CI: 0.8–3), and decreased during 2000–2002 (APC = -7.6, 95%CI: − 13.2--1.7), 2002–2010 (APC = -17.2, 95%CI: − 17.9--16.5) and 2017–2019 (APC = -26.3, 95%CI: − 30.8--21.5) (Fig. 3) (Table 1).

**Fig. 4:**
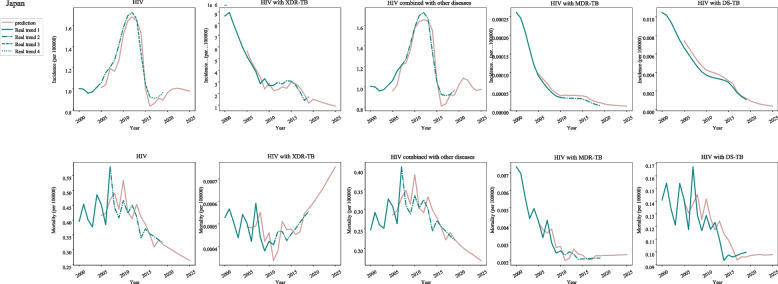
The predicted and actual incidence and mortality of HIV in Japan

In post-neonatal population from Japan, the incidences of HIV were increased during 2004–2012 (APC = 8.1, 95%CI: 7.2–9.1) and 2016–2019 (APC = 1.3, 95%CI: − 2-4.7), and decreased during 2000–2004 (APC = -0.5, 95%CI: − 2.5-1.6) and 2012–2016 (APC = -15.8, 95%CI: − 18.5--12.9). In HIV with XDR-TB, the incidences were increased during 2010–2015 (APC = 4.7, 95%CI: 3.8–5.7), and decreased during 2000–2002 (APC = -6.3, 95%CI: − 9.1--3.6), 2002–2010 (APC = -13.2, 95%CI: − 13.5--12.9) and 2015–2019 (APC = -18.4, 95%CI: − 19.1--17.6). For HIV accompanied with other diseases, increased incidences were observed during 2004–2012 (APC = 8.1, 95%CI: 7.2–9.1) and 2016–2019 (APC = 1.3, 95%CI: − 2-4.7) while decreased incidences were observed during 2000–2004 (APC = -0.5, 95%CI: − 2.5-1.6) and 2012–2016 (APC = -15.8, 95%CI: − 18.5--12.9). In terms of HIV with MDR-TB and DS-TB, the incidences were decreased during 2000–2019 (Fig. 4) (Table 1).

**Fig. 5:**
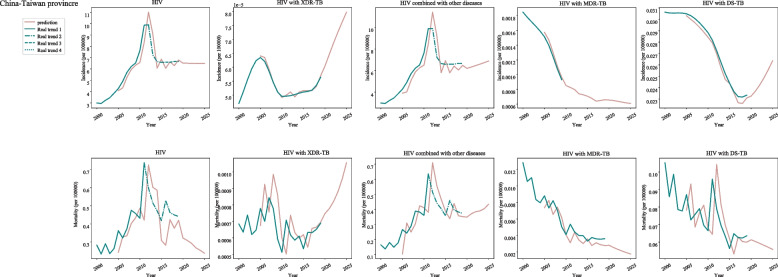
The predicted and actual incidence and mortality of HIV in Taiwan province

In post-neonatal population from Taiwan, the increased incidences of HIV were found during 2000–2012 (APC = 11.1, 95%CI: 9.8–12.4), and 2015–2019 (APC = 2.1, 95%CI: − 4-8.7), and decreased incidences were identified during 2012–2015 (APC = -12.8, 95%CI: − 28.3-6). The increased incidences of HIV with XDR-TB were observed during 2000–2005 (APC = 6.4, 95%CI: 5.8–7), 2010–2017(APC = 0.8, 95%CI: 0.3–1.2) and 2017–2019 (APC = 4.2, 95%CI: 1.5–7). The incidences of HIV combined with other diseases were increased during 2000–2012 (APC = 11.1, 95%CI: 9.8–12.4) and 2015–2019 (APC = 2.1, 95%CI: − 4-8.7). The incidences of HIV with MDR-TB were increased during 2017–2019 (APC = 0.5, 95%CI: − 0.8-1.8), and decreased during 2000–2017. For HIV with DS-TB, the incidence was decreased during 2000–2019 (APC = -2.3, 95%CI: − 3.1--1.4) (Fig. 5) (Table 1).

**Fig. 6:**
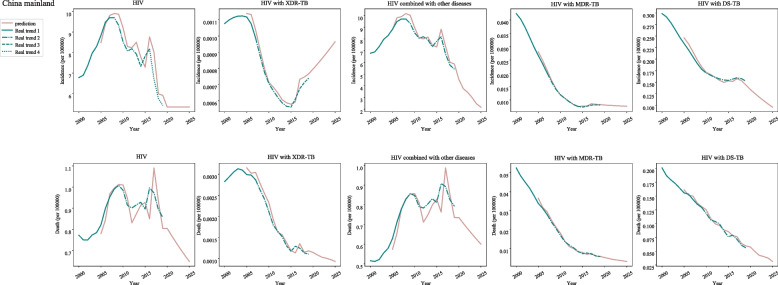
The predicted and actual incidence and mortality of HIV in China Mainland

As for the incidence of different types of HIV in post-neonatal population from China Mainland, increased incidences of HIV were identified during 2000–2007 (APC = 5.9, 95%CI: 4.8–7) and 2013–2016 (APC = 0.8, 95%CI: − 6.9-9.1), and decreased during 2007–2013 (APC = -4.2, 95%CI: − 5.9--2.5) and 2016–2019 (APC = -12.6, 95%CI: -16--9). The incidences of HIV with XDR-TB were elevated during 2000–2006 (APC = 0.1, 95%CI: − 0.7-0.9) and 2014–2019 (APC = 6.3, 95%CI: 5.1–7.4), and declined during 2006–2014. In terms of HIV combined with other diseases, the incidences were increased during 2000–2007 (APC = 5.9, 95%CI: 4.8–7) and 2013–2016 (APC = 0.8, 95%CI: − 6.9-9.1), and decreased during 2007–2013 (APC = -4.2, 95%CI: − 5.9--2.5) and 2016–2019. Decreased incidence of HIV with MDR-TB was found during 2000–2014, and then increased during 2014–2019 (APC = -12.6, 95%CI: -16--9). The incidence of HIV with DS-TB was decreased during 2000–2019 (Fig. 6) (Table 1).

### The mortality of HIV in post-neonatal population from East Asia during 2000–2019

In post-neonatal population from North Korea, the mortalities of HIV, HIV with XDR-TB, and HIV combined with other diseases were increased during 2000–2019. The mortality of HIV with MDR-TB was decreased during 2013–2019 (APC = -2.9, 95%CI: − 4.1--1.8) and HIV with DS-TB was decreased during 2012–2019 (APC = -4.2, 95%CI: − 4.6--3.8) (Fig. 1) (Table 1).

In terms of the mortality of post-neonatal population from South Korea, decreased mortality of HIV was observed during 2010–2019 (APC = -4.7, 95%CI: − 7.9--1.3). For HIV with XDR-TB, the mortality was decreased during 2004–2019 (APC = -2.9, 95%CI: − 4.2--1.5). The mortality of HIV combined with other diseases showed a decline during 2011–2019 (APC = -3.6, 95%CI: − 5.1--2.2). The mortality of HIV with MDR-TB was decreased during 2004–2009 (APC = -13.7, 95%CI: − 22.2--4.4) and 2009–2019 (APC = -6.0, 95%CI: − 8.3--3.6) while the mortality of HIV with DS-TB was decreased during 2010–2019 (APC = -4.5, 95%CI: − 7.5--1.4) (Fig. 2) (Table 1).

In post-neonatal population from Mongolia, the mortality of HIV was decreased during 2000–2007 and 2016–2019 (APC = -32.6, 95%CI: − 36.4--28.6). With regard to HIV with XDR-TB, the mortality was decreased during 2000–2010 (APC = -1.6, 95%CI: − 2.8--0.5) and 2016–2019 (APC = -28.3, 95%CI: − 33.7--22.6). Decreased mortality of HIV combined with other diseases was observed during 2003–2006 and 2016–2019 (APC = -32.7, 95%CI: − 36.6--28.5). The mortality of HIV with MDR-TB and HIV with DS-TB presented a decline during 2000–2010 and 2016–2019 (Fig. 3) (Table 1).

For post-neonatal population from Japan, during 2007–2019 (APC = -3.2, 95%CI: − 4.6--1.8), the mortality of HIV was decreased. During 2000–2011 (APC = -2.4, 95%CI: − 4.4--0.4), the mortality of HIV with XDR-TB was decreased, and during 2007–2019 (APC = -3.2, 95%CI: − 4.7--1.7), the mortality of HIV combined with other diseases were decreased. For HIV with MRD-TB and DS-TB, the mortality was decreased during 2000–2019 (Fig. 4) (Table 1).

In Taiwan, the mortality of HIV and HIV combined with other diseases in post-neonatal population was decreased during 2011–2019 (APC = -3.1, 95%CI: − 6.8-0.8). For HIV with XDR-TB, MDR-TB and DS-TB, a decline of mortality was observed during 2000–2019 (Fig. 5) (Table 1).

With regard to HIV in post-neonatal population from China Mainland, decreased mortality of HIV was found during 2008–2019 (APC = -0.6, 95%CI: − 1.6-0.3). As for the mortality of HIV with XDR-TB, the mortality showed a decline during 2007–2014 (APC = -11.2, 95%CI: − 13--9.3) and 2014–2019 (APC = -2.9, 95%CI: − 5.6--0.2). The mortality of HIV with MDR-TB and DS-TB was decreased during 2000–2019 (Fig. 6) (Table 1).

### The prediction of the incidence and mortality of HIV in post-neonatal population during 2020–2025

As for post-neonatal population in North Korea, the incidence of HIV was slowly increased from 2020 to 2025 while the mortality was decreased. For HIV with XDR-TB, both the incidence and mortality were rapidly increased during 2020–2025. The incidence of HIV combined with other diseases were increased during 2020–2024, but decreased in 2025. The mortality of HIV combined with other diseases was slowly decreased during 2020–2025. Increased incidence of HIV with MDR-TB was found during 2020–2025. The mortality of HIV with MDR-TB was slowly decreased during 2020–2025. The incidence and mortality of HIV with DS-TB was decreased in the next 5 years (Fig. 1) (Table 2).

**Table 2:** The prediction of the incidence and mortality of HIV in post-neonatal population during 2020–2025

		The predicted value in 2020–2025	MAE	MSE	RMSE			The predicted value in 2020–2025	MAE	MSE	RMSE
North Korea											
Incidence	HIV	20.531	0.270	0.111	0.334	Mortality	HIV	2.584	0.086	0.008	0.090
		20.806						2.555			
		21.076						2.511			
		21.341						2.452			
		21.601						2.378			
	XDR-TB	0.0037	< 0.001	< 0.001	< 0.001		XDR-TB	0.0059	< 0.001	< 0.001	< 0.001
		0.0039						0.0062			
		0.0042						0.0065			
		0.0045						0.0067			
		0.0048						0.0071			
	Combined with other diseases	20.883	0.402	0.209	0.456		Combined with other diseases	2.151	0.019	< 0.001	0.0120
		21.366						2.125			
		21.992						2.093			
		22.733						2.052			
		23.622						2.004			
	MDR-TB	0.039	0.001	< 0.001	0.001		MDR-TB	0.0318	< 0.001	< 0.001	< 0.001
		0.040						0.0318			
		0.041						0.0317			
		0.042						0.0315			
		0.043						0.0312			
	DS-TB	0.604	0.009	< 0.001	0.011		DS-TB	0.236	0.026	< 0.001	0.026
		0.657						0.229			
		0.535						0.223			
		0.496						0.216			
		0.455						0.210			
South Korea											
Incidence	HIV	7.317	0.339	0.149	0.386	Mortality	HIV	0.328	0.012	< 0.001	0.015
		8.442						0.314			
		10.035						0.303			
		12.528						0.293			
		16.180						0.286			
	XDR-TB	0.00011	< 0.001	< 0.001	< 0.001		XDR-TB	0.00146	< 0.001	< 0.001	< 0.001
		0.00010						0.00146			
		0.00009						0.00147			
		0.00009						0.00147			
		0.000085						0.00147			
	Combined with other diseases	7.055	0.371	0.215	0.464		Combined with other diseases	0.173	0.015	<.001	0.01
		8.179						0.165			
		9.772						0.158			
		12.265						0.153			
		15.917						0.148			
	MDR-TB	0.000851	< 0.001	< 0.001	< 0.001		MDR-TB	0.00477	< 0.001	< 0.001	< 0.001
		0.000762						0.00455			
		0.000684						0.00434			
		0.000614						0.00415			
		0.000552						0.00396			
	DS-TB	0.039	0.004	< 0.001	0.005		DS-TB	0.130	0.010	< 0.001	0.010
		0.031						0.120			
		0.024						0.113			
		0.017						0.105			
		0.011						0.098			
Mongolia											
Incidence	HIV	0.677	0.192	0.049	0.222	Mortality	HIV	0.037	0.036	0.002	0.046
		0.587						0.020			
		0.505						0.0089			
		0.433						0.002			
		0.340						0.000			
	XDR-TB	0.00006	< 0.001	< 0.001	< 0.001		XDR-TB	0.0001	< 0.001	< 0.001	< 0.001
		0.00003						0.00003			
		0.00005						0.00001			
		0.000004						0.000007			
		0.000						0.000006			
	Combined with other diseases	0.835	0.066	0.006	0.078		Combined with other diseases	0.0365	0.034	0.001	0.0433
		0.807						0.0205			
		0.780						0.0100			
		0.754						0.003			
		0.728						0.000			
	MDR-TB	0.00024	< 0.001	< 0.001	< 0.001		MDR-TB	0.0004	< 0.001	< 0.001	< 0.001
		0.00012						0.0003			
		0.000050						0.0002			
		0.000009						0.0001			
		0.000						0.00008			
	DS-TB	0.00379	0.001	< 0.001	0.002		DS-TB	0.00288	0.002	< 0.001	0.002
		0.00288						0.00255			
		0.00255						0.00247			
		0.00247						0.00244			
		0.00244						0.00288			
Japan											
Incidence	HIV	0.996	0.106	0.026	0.163	Mortality	HIV	0.299	0.026	0.001	0.029
		1.006						0.289			
		1.001						0.281			
		0.989						0.272			
		0.979						0.264			
	XDR-TB	0.0000014	< 0.001	< 0.001	< 0.001		XDR-TB	0.00062	< 0.001	< 0.001	< 0.001
		0.0000012						0.00065			
		0.0000011						0.00069			
		0.0000010						0.00072			
		0.0000009						0.00075			
	Combined with other diseases	1.083	0.112	0.024	0.155		Combined with other diseases	0.201	0.017	< 0.001	0.021
		1.062						0.192			
		0.995						0.185			
		0.961						0.177			
		0.973						0.169			
	MDR-TB	0.0000139	< 0.001	< 0.001	< 0.001		MDR-TB	0.0018.	< 0.001	< 0.001	< 0.001
		0.0000126						0.00181			
		0.0000116						0.00182			
		0.0000109						0.00183			
		0.0000104						0.00184			
	DS-TB	0.000744	< 0.001	< 0.001	< 0.001		DS-TB	0.0975	0.005	< 0.001	0.007
		0.000609						0.0980			
		0.000508						0.0974			
		0.000432						0.0976			
		0.000374						0.0978			
Taiwan											
Incidence	HIV	6.508	0.224	0.074	0.272		HIV	0.309	0.11	0.013	0.115
		6.494						0.293			
		6.484						0.268			
		6.481						0.254			
		6.482						0.238			
	XDR-TB	0.000065	< 0.001	< 0.001	< 0.001		XDR-TB	0.00078	< 0.001	< 0.001	< 0.001
		0.000069						0.00082			
		0.000072						0.00087			
		0.000076						0.00096			
		0.000079						0.00106			
	Combined with other diseases	6.326	0.403	0.212	0.460		Combined with other diseases	0.370	0.025	< 0.001	0.026
		6.456						0.385			
		6.592				Mortality		0.390			
		6.734						0.404			
		6.880						0.434			
	MDR-TB	0.000649	< 0.001	< 0.001	< 0.001		MDR-TB	0.00256	< 0.001	< 0.001	< 0.001
		0.000638						0.00233			
		0.000627						0.00212			
		0.000617						0.00194			
		0.000609						0.00176			
	DS-TB	0.00237	< 0.001	< 0.001	< 0.001		DS-TB	0.0603	0.002	< 0.001	0.002
		0.00242						0.0592			
		0.00249						0.0582			
		0.00256						0.0570			
		0.00264						0.0558			
China mainland											
Incidence	HIV	5.345	0.350	0.243	0.493	Mortality	HIV	0.770	0.054	0.004	0.062
		5.345						0.738			
		5.345						0.707			
		5.345						0.677			
		5.345						0.648			
	XDR-TB	0.00082	< 0.001	< 0.001	< 0.001		XDR-TB	0.00109	< 0.001	< 0.001	< 0.001
		0.00085						0.00103			
		0.00089						0.00101			
		0.00093						0.000978			
		0.00096						0.000934			
	Combined with other diseases	3.758	0.580	0.482	0.694		Combined with other diseases	0.707	0.053	0.004	0.060
		3.448						0.679			
		2.995						0.651			
		2.462						0.625			
		2.179						0.600			
	MDR-TB	0.00799	< 0.001	< 0.001	< 0.001		MDR-TB	0.0046	< 0.001	< 0.001	0.01
		0.00789						0.0042			
		0.00780						0.0039			
		0.00772						0.0036			
		0.00763						0.0032			
	DS-TB	0.126	0.007	< 0.001	0.009		DS-TB	0.0496	0.003	< 0.001	0.004
		0.118						0.0425			
		0.110						0.0403			
		0.103						0.0375			
		0.096						0.0313			

*MAE* Mean absolute error, *MSE* The mean squared error, *RMSE* Root mean square error, *XDR-TB* Extensive drug-resistant tuberculosis, *MDR-TB* Multidrug-resistant TB, *DS-TB* Drug-susceptible TB

As shown in Table 2, the incidence of HIV in post-neonatal population from South Korea was rapidly increased and the mortality was decreased during 2020–2025. The incidence of HIV with XDR-TB was decreased during 2020–2025 while the mortality was stationary during 2020–2021, and increased during 2022 and then stationary to 2025. A rapid increase of incidence and slowly decrease of mortality in HIV with other diseases were observed during 2020–2025. Both the incidence and mortality in HIV with MDR-TB and DS-TB presented a declined trend (Fig. 2) (Table 2).

In terms of the predicted incidence and mortality in post-neonatal population from Mongolia, decreased incidence and mortality of HIV, HIV with XDR-TB, HIV combined with other diseases, HIV with MDR-TB and DS-TB was observed during 2020–2025 (Fig. 3) (Table 2).

In Japan, the predicted incidence of HIV was increased during 2020–2021 and then slowly decreased during 2022–2025. The mortality was decreased during 2020–2025. A decreased incidence and increased mortality of HIV with XDR-TB was identified during 2020–2025. The incidence of HIV combined with other diseases was decreased during 2020–2024 but increased in 2025. The mortality of HIV combined with other diseases showed rapid decline during 2020–2025. The incidence of HIV with MDR-TB showed a slow decrease while the mortality of HIV with MDR-TB exhibited a slow increase. In terms of DS-TB, the incidence was slowly decreased while the mortality was increased during 2020–2021 and decreased during 2022–2025 (Fig. 4) (Table 2).

With regard to the post-neonatal population from Taiwan, the incidence of HIV showed a decreased trend during 2020–2024 and was increased slowly in 2025 while the mortality of HIV was quickly decreased during 2020–2025. Rapid increase of the incidence and mortality of HIV with XDR-TB was observed during 2020–2025. As for the incidence and mortality of HIV combined with other diseases, a slow increase was identified during 2020–2025. The incidence and morality of HIV with MDR-TB was gradually declined during 2020–2025. In terms of HIV with DS-TB, the incidence presented a quickly increase trend while the mortality displayed a gradual decreased trend during 2020–2025 (Fig. 5) (Table 2).

In China mainland, the incidence of HIV in post-neonatal population was similar while the mortality of HIV decreased slowly during 2020–2025. Rapid increase was observed in the incidence of HIV with XDR-TB during 2020–2022. The incidence and mortality of HIV combined with other diseases, HIV with MDR-TB and HIV with DS-TB exhibited a decreased trend during 2020–2025 (Fig. 6) (Table 2).

## Discussion

In the present study, we evaluated the incidence and mortality of HIV in post-neonatal population from East Asia during 2000–2019 based on the data from GBD and predicted the incidence and mortality of HIV in the population during 2020–2025. In North Korea, the incidence and mortality of HIV showed a rapid increase during 2000–2010 and a gradual decrease during 2010–2019. The incidence of HIV was predicted to be increased and the mortality was decreased. In South Korea, the incidence during 2000–2010 was increased and decreased during 2010–2019, while the mortality showed fluctuant trend. As predicted, the incidence of HIV in South Korea might be increased and the mortality might be decreased during 2020–2025. In Mongolia, the incidence and mortality were slowly decreased during 2000–2005, increased during 2005–2015, and then rapidly decreased till 2019. The predicted incidence and mortality of HIV showed a decreased trend. As for Japan, the incidence of HIV was rapidly increased till 2010 and then decreased till 2015. The predicted incidence of HIV in Japan was gradually increased. The mortality of HIV in Japan was fluctuant during 2000–2019 and was slowly decreased as predicted. The incidence and mortality of HIV in Taiwan during 2000–2019 was increased on the whole. The predicted incidence of HIV during was stationary and the mortality was decreased. In terms of China Mainland, the incidence and mortality of HIV was fluctuant during 2000–2019. The predicted incidence of HIV in China Mainland was stationary while the mortality was rapidly decreased.

According to the results in this study, the incidence of HIV in post-neonatal population was increased on the whole in East Asia during 2000–2019. This may be due to the remarkable progress achieved in the diagnosis of HIV, especially the increase for the diagnosis of HIV infection in 20s and 30s women who at reproductive age [29]. During 2010–2015, the incidence of HIV showed a decreased trend. The World Health Organization (WHO) provided and updated the recommendations on the regimens of antiretrovirals (ARVs) for pregnant women infected with HIV [30–32]. Additionally, some East Asia countries launched the prevention of mother-to-child transmission for HIV program and spread more knowledge of HIV transmission means from mother to child among women [33, 34]. The predicted incidence of HIV in post-neonatal population showed a decreased trend in East Asia, except North Korea and South Korea. This might because in South Korea, the incidence of HIV has been steadily on the rise, especially among adolescents (ages 10–19 years) and young adults (ages 20–29 years) [29, 35]. As antiretroviral therapy (ART) has increased the life expectancy of HIV patients, women HIV patients are now more frequently considering becoming pregnant [36]. The live birth rate in women HIV patients is nearly as high as women without HIV infection with the application of ART [37]. Additionally, the repeated pregnancies among women with HIV was also increased in recent years [38, 39]. These factors might be the reason for the increase of HIV incidence post-neonatal population. In the current study, the mortality of HIV in post-neonatal population in East Asia was decreased since 2010 and the predicted mortality during 2020–2025 was continued to decline. This maybe because the improved prevention, screening, and treatment strategies including ART for HIV [40, 41]. Early HIV screening tests, avoid breastfeeding, and health care were provided in post-neonatal population with HIV, which might also help decrease the mortality of these patients [33]. In general, the incidence of HIV with XDR-TB in South Korea, North Korea, Mongolia, and Taiwan was increased during 2000–2019. According to the data from Zhang et al., the incidence rate of HIV with XDR-TB in all population from East Asia were increased from 1990 to 2017 [42]. The increased number of women HIV infection might be associated with the elevated incidence of post-neonatal HIV infection. As for North Korea, China Mainland and Taiwan, the increased incidence trend of HIV with XDR-TB was found during 2020–2025, and more effective measures should be taken to prevent the occurrence of HIV with XDR-TB. Meanwhile, the mortality of post-neonatal HIV in most East Asia countries and territories were decreased from 2000 to 2019 except North Korea in our study. The decreasing tendency of mortality indicates the great improvements in HIV treatments due to the introduction of ART [43]. It is worth noting that the mortality of post-neonatal HIV in North Korea and Japan during 2020–2025 presented an increasing tendency. The management of HIV with XDR-TB is more complicated and early use of ART and early testing to the drug resistance status should be provided to improve the outcomes of these patients.

On the whole, the trend of incidence of HIV combined with other diseases in post-neonatal population was increased before 2010 followed by decrease during 2010–2019. Infants who infected with HIV through MTCT have high possibility of combining with other diseases since born. A previous study on evaluating the outcomes of neonates whose mother were infected with HIV, and the results demonstrated that most neonates were born prematurely and associated with low birth weight [44]. This was supported by several studies in other countries, including a preterm birth rate of 13.0% in the United Kingdom and a 23.7% preterm birth rate, and an 18.4% small for gestational age rate in Botswana [45, 46]. Preterm or low birth weight neonates were reported to combine with many diseases [47, 48]. The predicted incidence of HIV combined with other diseases in post-neonatal population was increased in North Korea, South Korea and Taiwan during 2020–2025. Increased incidence of HIV in post-neonatal population might result in a higher rate of those patients combined with other diseases. More care should be provided on puerpera infected with HIV and their infants to prevent the occurrence of HIV or other diseases on the babies. The overall mortality of HIV combined with other diseases in post-neonatal population began to decrease in recent years from East Asia. As the increase of the knowledge on HIV, timely diagnosis and treatments were provided to infants with HIV, which helped improve the outcomes of those patients. On the whole, the incidence and mortality of HIV with MDR-TB and DS-TB in post-neonatal population from East Asia showed a decreased trend. A mathematical model constructed by Cho et al. for predicting the TB burden in South and North Korea delineated that the control strategies of TB including latent TB infection treatment, rapid diagnosis, active case-finding and improvement of the treatment success rate would reduce the burden of TB [49]. Zhang et al. identified that the overall mortality in all population with HIV and TB co-infection was decreased in East Asia in recent years [42]. These gave evidence to the finding of our study.

In previous studies, multiple evidence exhibited that the establishment of a single model presented a good effect when dealing with a single time series forecasting problem, but limitations existed when facing the complex problems [50, 55

Artificial intelligence (AI) is novel method to solve complex problems using computer engineering and software development [56]. As one of the applications of AI, ML provide output to almost any given input based on training, which ultimately improves the predictive value for a given task and/or acquire new skills over time when trained with more data [15]. In recent years, multiple ML, and AI methods were applied for helping predict ground water storage change [11], climate [12, 13] et al., and more and more studies indicated that artificial neural networks were more robust than linear regression as they more efficiently processed large datasets, worked with incomplete data, handled both linear and nonlinear processes and identified more relationships not inputted by the user [57]. In our study, we used LSTM to predict the HIV incidence and mortality of post-neonatal population in East Asia. Dropouts and callbacks were used to prevent the overfitting in the LSTM model. The results might help guide the clinicians and governments to make further policies for HIV prevention and treatment in both adults and neonatal.

This study evaluated the incidence and mortality of HIV in post-neonatal population based on the data from GBD, the data from GBD are authoritative and reliable. We were focused on HIV in post-neonatal population, and the results of our study might help decrease the occurrence of HIV and improve the outcome in those population, which might decrease the burden of society. There were several limitations of our study. Firstly, the data from GBD was updated to 2019, the recent data on 2020 was still unavailable to validate the results in the present study. Secondly, the prediction of incidence and mortality was only evaluated according to the time series, if other variables were included, the results of our study might be more reliable. In the future, we will update our prediction through using the recent data on 2020–2022, and the actual incidence and mortality of HIV in post-neonatal population could be compared with the predicting results in our study to validate the prediction value of our model. Also, we will apply more ML and DL methods to improve the predictive accuracy for the incidence and mortality of HIV in post-neonatal population, and hope to provide a reference for the government and clinicians to make future polices in the prevention and treatments of HIV.

## Conclusions

The incidence and mortality of HIV in post-neonatal population from East Asia during 2000–2019 was evaluated and the incidence and mortality of HIV in the population during 2020–2025 was predicted in the current study. On the whole, the incidence of HIV combined with other diseases in post-neonatal population was increased before 2010 and then decreased during 2010–2019 while the mortality of those patients was decreased in East Asia. Strategies should be applied in respective countries to decrease the incidence and mortality of HIV in those population.

## Data Availability

The datasets used and/or analyzed during the current study are available from the corresponding author on reasonable request.

## Abbreviations

HIV: Human immunodeficiency virus
MTCT: Mother-to-child transmission
UNAIDS: The Joint United Nations Program on HIV/AIDS
ARIMA: Autoregressive integrated moving average
LSTM: Long short-term memory
GBD: Global Burden of Disease
YLLs: Years of life lost
YLDs: Years lived with disability
DALYs: Disability-adjusted life-years
APC: Annual percent changes
XDR-TB: Extensive drug-resistant tuberculosis
DS-TB: Drug-susceptible TB
